# Asthma policy in Illinois: A survey of school nursing and staff knowledge and implementation patterns

**DOI:** 10.1111/phn.13337

**Published:** 2024-05-15

**Authors:** Paige Hardy, Michael Gonzalez, Anna Volerman, Erica Salem, Nancy Amerson, Nikki Woolverton, Sarah Dee Geiger, Andrea A. Pappalardo

**Affiliations:** 1 Department of Medicine, University of Illinois Chicago, Chicago, Illinois, USA; 2 Department of Pediatrics, University of Illinois Chicago, Chicago, USA; 3 Strategy, Programs & Policy, Respiratory Health Association, Chicago, Illinois, USA; 4 Illinois Department of Public Health, Springfield, Illinois, USA; 5 Department of Kinesiology and Community Health, University of Illinois at Urbana-Champaign, Urbana-Champaign, Illinois, USA

**Keywords:** asthma, health policy, implementation science, school health services, school nursing, schools

## Abstract

**Objective:**

Our goal is to examine gaps in self-carry, asthma emergency protocol, and stock inhaler policy knowledge in Illinois schools.

**Design:**

A 30-item REDCap cross-sectional survey developed by a team of stakeholders was disseminated. Questions assessed policy knowledge, awareness, and practices regarding asthma emergency protocols, self-carry, and stock inhalers.

**Sample:**

Participants were Illinois school nurses belonging to a governmental organization listserv.

**Measurements:**

Analysis utilized Chi-square tests, descriptive statistics, and *t*-tests.

**Results:**

Nurses reported 36% of students on average self-carried asthma medication. Thirty percent of nurses were not aware of their emergency asthma policy and only 60% reported having an emergency asthma protocol in their school(s). Fifty-four percent of nurses were aware of stock inhaler programming. Of the 10.3% who reported a stock inhaler program, a lower frequency reported calling 911 for asthma emergencies. Perceived school asthma prevalence varied from 0%–87%.

**Conclusions:**

Our survey demonstrates large variation in knowledge and implementation of school-based asthma health policy. This is likely due to variations in health policy education dissemination. Future efforts should focus on the dissemination and implementation of school-based asthma health policies to improve their more universal adoption and better support school-based asthma management.

## 1 | BACKGROUND

Six and a half million children in the United States (US) have asthma, accounting for 790,000 emergency room visits and at least $272 million in emergency department Medicaid costs in 2010 (Pearson et al., 2014). US children with asthma collectively missed more than 13 million school days in 2013, compromising their education and future earning potential (O’Rourke et al., 2020). Worse, 44% of US children with asthma had uncontrolled asthma, according to data collected between 2018–2020 (Centers for Disease Control and Prevention, 2022). Health policies can be used to create systematic approaches toward childhood asthma management. Existing policies have been shown to directly benefit children regardless of their background, especially in the school setting (Hester et al., 2013). Regarding asthma, Illinois passed a 2001 self-carry law, an asthma emergency protocol mandate in 2016, and a 2018 undesignated asthma medication law (sometimes called a stock inhaler law) (Illinois General Assembly, 2023a, 2023b, 2023c). Self-carry or self-medication laws allow children to keep their medication on their person and administer it whenever necessary (Illinois General Assembly, 2023a). Asthma emergency protocols outline what school nurses and other staff must do in the case of asthma emergency (Illinois General Assembly, 2023b). Additionally, stock inhaler laws enable schools to provide an undesignated asthma inhaler to anyone experiencing respiratory distress on school grounds (Illinois General Assembly, 2023c). All these policies enable schools to better manage asthma symptoms, especially considering that triggers are common in school settings (Hauptman & Phipatanakul, 2015).

According to the 2018 National School Nurse Workforce Study, 4.8% of schools in the midwestern region of the US have at least one Licensed Practice Nurse (LPN), 64.5% have at least one Registered Nurse (RN), and 6.9% have both. More than two thirds (67.2%) of Midwestern nurses work in two or more schools (Volerman et al., 2021). However, stock inhaler program uptake in Illinois has been slow, with only 11 districts reporting stock inhaler use over the 2021–2022 school year (Ayala, 2022). This is a fraction of the 1,942,899 students Illinois serviced in 851 public school districts over the 2019–2020 school year (Ilinois State Board of Education, 2023).

Health policy is vital to guide chronic condition management but can be difficult to implement widely and with fidelity, preventing these policies from achieving their fullest potential (Willgerodt et al., 2018). Despite having a health policy called the “Managing Asthma in Schools Initiative,” New York City teachers felt that lack of policy knowledge prevented them from adequately managing asthma in their classrooms (Cain & Reznik, 2016). Although this highlights the critical need to monitor asthma health policy adoption, health policy evaluation data in asthma are limited for all stages of implementation (Willgerodt et al., 2018). Therefore, our goal was to examine gaps in asthma health policy knowledge and implementation in Illinois schools by surveying school nurses statewide. School nurses were targeted because they tend to be experts regarding individual school health protocols and procedures. We hypothesized that knowledge of asthma health policies is associated with implementation. Our secondary aim was to establish infrastructure for future statewide health policy evaluation.

## 2 | METHODS

### 2.1 | Design

This survey was created and funded by a government agency and a lung health advocacy association to ascertain policy knowledge and implementation among school nurses. The survey was exempt from IRB approval because it was conceived and executed as a quality improvement project through a state health department for public health activities. Academic researchers including asthma subspecialists, community-based researchers, and representatives from a professional school nursing organization were consulted in the construction of a 30-item cross-sectional survey in REDCap and to faciliate the dissemination of findings, and the agencies themselves administered the survey and performed the analysis.

#### Sample:

The survey was disseminated electronically to 2555 Professional Educator License (PEL) certified and non-PEL nurses in Illinois via an email listserv. The survey was open from August 2020 to September 2020. One reminder email was sent to encourage survey participation. Demographic data was not collected.

### 2.2 | Measurements

Survey questions assessed perceived asthma prevalence, asthma policy knowledge, confidence in managing asthma, effectiveness of existing policy, and barriers to school-based asthma care.

### 2.3 | Analytic strategy

Data were analyzed in Excel using Chi-Squared analysis, student’s *t*-tests, and descriptive statistics.

## 3 | RESULTS

Of the 2555 school nurses on the IDPH school health program listserv, 8% (*n* = 204) responded, representing 85 public school districts, nine nonpublic schools, and 29 unknown institutions. Sixty-three percent of nurses worked in one school only, 13% in two schools, and 38% in three or more schools. The perceived prevalence of asthma among children in their school(s) varied, with an average of 21%, although there was a large range (0%–87%). Over 90% of nurses reported that they were confident in treating asthma.

**FIGURE 1:**
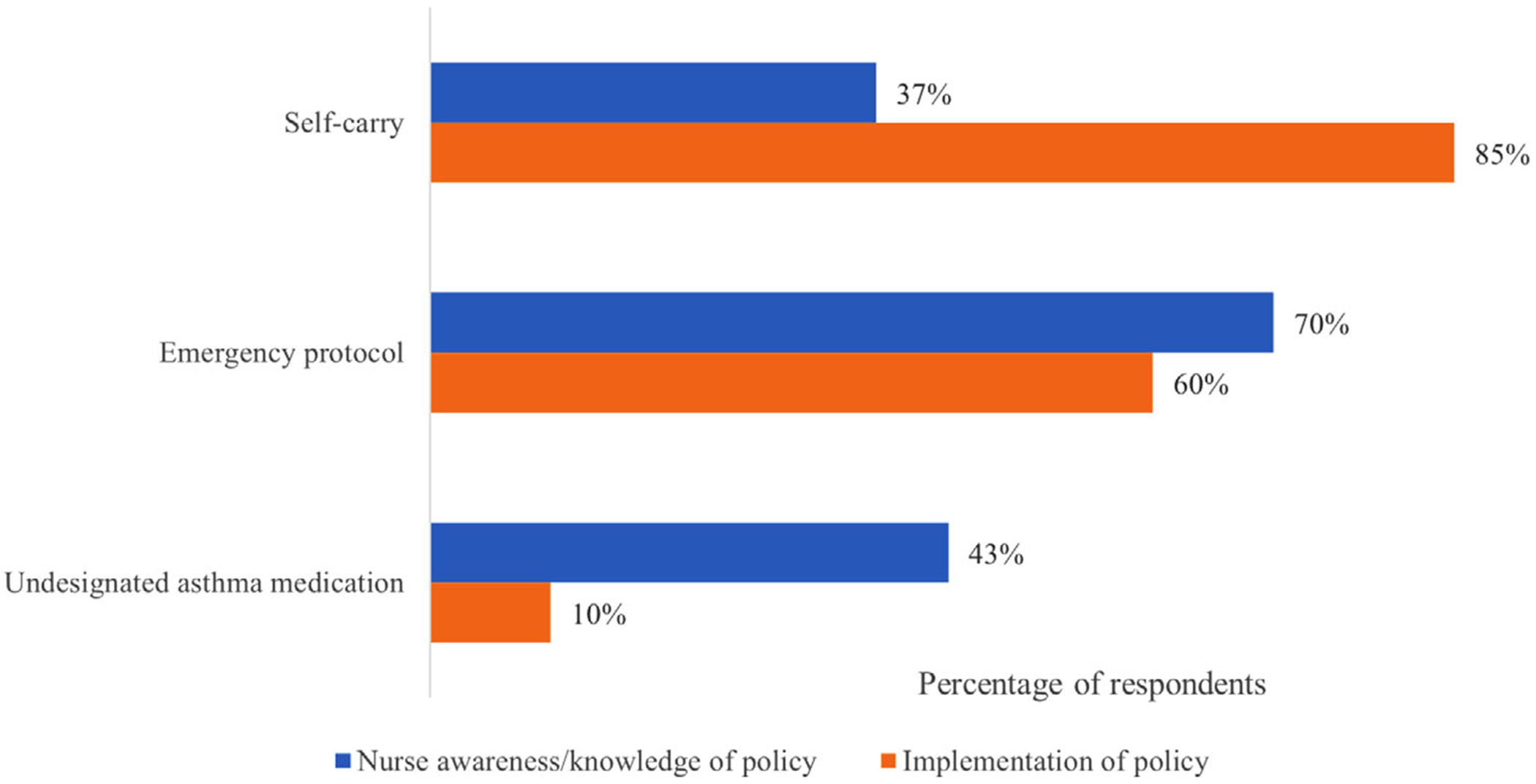
This figure displays the frequency of ways nurse respondents have initially learned about their student’s asthma. Nurses were able to select multiple options. The most common methods were notification by parents and school physical forms, with over four out of every five respondents selecting each answer. Over half of nurses (52%) had learned of a student’s asthma diagnosis when treating them in the nurse’s office, and 27% reported recognizing asthma during an emergency. [Color figure can be viewed at wileyonlinelibrary.com]

More than half (52%) of nurses surveyed had learned of students’ asthma diagnoses during a respiratory event at school, including during an emergency episode on school grounds (Figure 1). Almost all (95%) reported requesting Asthma Action Plans (AAPs) from caregivers and had elicited them in numerous ways, most often including letters (83%) and emails (71%). Despite this effort, nurses reported, on average, only about half (49%) of students with asthma had a complete AAP on file with the school.

Most nurses surveyed (80%) felt students with asthma benefit from the presence of a self-carry and self-administration asthma policy. However, only 36% of students with asthma had permission to self-carry their asthma medication.

**FIGURE 2:**
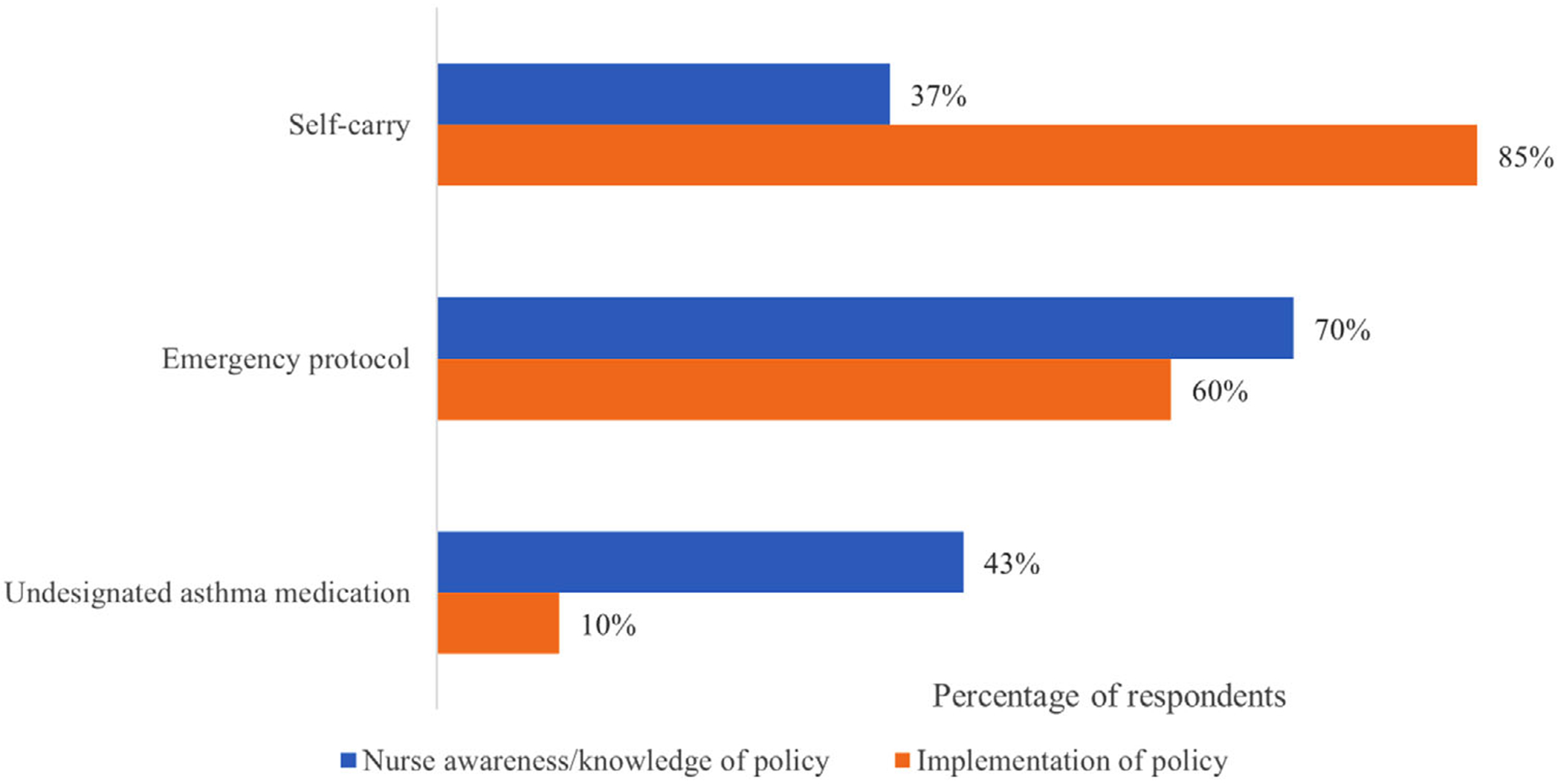
This figure displays how many nurse respondents were aware of important self-carry, emergency protocol, and undesignated asthma medication policy requirements in blue and how many had implemented these policies within their schools in orange. Knowledge of self-carry polices was evaluated by asking nurses whether written authorization documentation needed to be submitted for students to bring and self-administer their asthma medication at school. Asthma Emergency Protocol awareness was evaluated by asking nurses if they were aware of the state requirement to develop an Asthma Emergency Protocol and implementation through asking if they had read or reviewed their school’s protocol. Undesignated asthma medication knowledge was determined through a question concerning the ability for any trained personnel to administer the undesignated inhaler. [Color figure can be viewed at wileyonlinelibrary.com]

Eighty-six percent of nurses correctly responded that parental permission is required for children to self-carry and administer their inhaler, but only 37% correctly responded that signed permission from a medical provider was not required (Figure 2). We saw a statistically significant positive correlation between the correct response and higher reported percentage of self-carry students (*p* = .013).

Illinois health policy requires all districts to maintain a standard asthma-related emergency protocol. Only 60% of school nurses reported having an emergency action protocol in their school(s) (Figure 2) Thirty percent of respondents had either not read or were unaware of their policy. Half (50%) of nurses were aware that asthma emergency protocol training is required every 2 years. School nurses that reported monitored compliance with emergency protocol training were significantly more likely to be aware of this training requirement (*p* = .004).

Fifty-four percent of nurses were aware of undesignated asthma medication legislation (stock inhalers) and 10% reported having an implemented undesignated asthma medication program in their school (Figure 2). Significantly fewer nurses with undesignated medication reported calling 911 for an asthma emergency between the 2018 and 2020 school year compared to those without (*p* = .009).

## 4 | DISCUSSION

We discovered connections between nurses’ lack of policy awareness and lower implementation of asthma health policy, emphasizing the importance of disseminating knowledge of and implementation strategies for health policies after achieving legislative approval. Knowledge of emergency asthma protocol and self-carry policies were correlated with implementation. Asthma self-management education has been shown to be effective in managing asthma and improving clinical outcomes (Volerman et al., 2020). We recommend further education for nurses regarding the three policies be prioritized.

One area this improved policy knowledge can directly affect desired asthma outcomes is through increased implementation of stock inhaler programs, which have been shown to be effective in allowing students to return to class (Gerald et al., 2016). Since a high frequency of primary asthma events happens in schools, and students who self-carry may forget to bring their inhalers, implementation of undesignated asthma medication is a crucial part of school-based asthma management.

We also observed that surveyed nurses perceive a higher average prevalence of asthma in their schools (20%) than IDPH reports as the childhood asthma prevalence in Illinois (13%) (Centers for Disease Control and Prevention, 2022). More research is needed to show whether this perceived prevalence reflects underdiagnosis of asthma in Illinois children or further need for school-based asthma management support. Another major finding was that a large percentage of nurses encountered students in respiratory distress in students without a known diagnosis of asthma. This supports the idea that stock inhaler policies can be not only a failsafe option for children with undiagnosed asthma to de-escalate emergencies when they present in school, but also the utilization and documentation of the use of stock inhalers may also encourage asthma diagnosis to be formalized (Volerman et al., 2020). Regardless, we must act to ensure that schools are equipped to deal with asthma emergencies and management on a daily basis by having a school nurse in every school, as recommended by the National Association of School Nurses.

Our cross-sectional study had some limitations. We had a low response rate (8%) and likely had a response bias- nurses serving only one school may have had more time to take the survey and therefore been overrepresented in our dataset, potentially explaining why only 32% of nurses in our survey served two or more schools, but 67.2% of National School Nurse Workforce Study participants did (Volerman et al., 2021). Demographic information was not collected, so we cannot speak to the representativeness of our sample in terms of race, ethnicity, sex, or geographic location.

It is apparent that school nurses prioritize student health but are unable to adequately administer care without consistent access to trained staff, policy education and resources. This study provides vital data to elucidate the public health intersection of school staff knowledge, policy implementation, and asthma outcomes. Through these, we can construct targeted efforts to decode implementation barriers impeding asthma management and ultimately improve the health of our school-aged children with asthma.

## Data Availability

The data that support the findings of this study are available from the corresponding author upon reasonable request.

## References

[R1] Ayala C (2022). The administration of undesignated asthma medication, School year 2021–2022. Retrieved December 5, 2023 from https://www.ilga.gov/reports/ReportsSubmitted/3590RSGAEmail7221RSGAAttachThe%20Administration%20of%20Undesignated%20Asthma%20Medication%20School%20Year%202021-22.pdf

[R2] Cain A, & Reznik M (2016). Asthma management in New York City schools: A classroom teacher perspective. Journal of Asthma, 53(7), 744–750. 10.3109/02770903.2015.1135946 PMC4992552 27031532

[R3] Centers for Disease Control and Prevention. (2022). Uncontrolled Asthma Among Children With Current Asthma, 2018–2020. Retrieved December 5, 2023 from https://www.cdc.gov/asthma/asthma_stats/uncontrolled-asthma-children-2018-2020.htm

[R4] Gerald LB, Snyder A, Disney J, Gerald JK, Thomas A, Wilcox G, & Brown MA (2016). Implementation and Evaluation of a Stock Albuterol Program for Students with Asthma. Annals of the American Thoracic Society, 13(2), 295–296. 10.1513/AnnalsATS.201510-683LE 26848605

[R5] Hauptman M, & Phipatanakul W (2015). The school environment and asthma in childhood. Asthma Research and Practice, 1(1), 12. 10.1186/s40733-015-0010-6 26523228 PMC4627718

[R6] Hester LL, Wilce MA, Gill SA, Disler SL, Collins P, & Crawford G (2013). Roles of the State Asthma Program in Implementing Multicomponent, School-Based Asthma Interventions. Journal of School Health, 83(12), 833–841. 10.1111/josh.12101 24261517 PMC4555870

[R7] Illinois General Assembly (N.D.). Illinois Public Act 092–0402 [Law]. Retrieved December 5, 2023 from https://ilga.gov/legislation/ilcs/fulltext.asp?DocName=010500050K22-30#:~:text=Sec.,asthma%20episode%20emergency%20response%20protocol

[R8] Illinois General Assembly (N.D.). Illinois Public Act 099–0843 [Law]. Retrieved December 5, 2023 from https://ilga.gov/legislation/publicacts/fulltext.asp?name=099-0843

[R9] Illinois General Assembly (N.D.). Illinois Public Act 100–0726 [Law]. Retrieved December 5, 2023 from https://ilga.gov/legislation/publicacts/fulltext.asp?name=100-0726

[R10] Illinois State Board of Education (N.D). Annual statistical report 2019–2020 [Data set]. Retrieved June 21, 2023 from https://www.isbe.net/Pages/Annual-Statistical-Report.aspx

[R11] O’Rourke A, Zimmerman A, Platt H, & Pappalardo AA (2020). Preventing Asthma Emergencies in Schools. Pediatrics, 145(4), e20191995. 10.1542/peds.2019-1995 32193211

[R12] Pearson WS, Goates SA, Harrykissoon SD, & Miller SA (2014). State-Based Medicaid Costs for Pediatric Asthma Emergency Department Visits. Preventing Chronic Disease, 11, 140139. 10.5888/pcd11.140139 PMC4075488 24967830

[R13] Volerman A, Lowe AA, Pappalardo AA, Anderson CMC, Blake KV, Bryant-Stephens T, Carr T, Carter H, Cicutto L, Gerald JK, Miller T, Moore NS, Phan H, Sadreameli SC, Tanner A, Winders TA, & Gerald LB (2021). Ensuring Access to Albuterol in Schools: From Policy to Implementation. An Official ATS/AANMA/ALA/NASN Policy Statement. American Journal of Respiratory and Critical Care Medicine, 204(5), 508–522. 10.1164/rccm.202106-1550ST 34499024 PMC8491259

[R14] Volerman A, Kim TY, Sridharan G, Toups M, Hull A, Ignoffo S, Sharp LK, & Press VG (2020). A Mixed-methods Study Examining Inhaler Carry and Use among Children at School. Journal of Asthma, 57(10), 1071–1082. 10.1080/02770903.2019.1640729 PMC6962574 31274042

[R15] Willgerodt MA, Brock DM, & Maughan ED (2018). Public School Nursing Practice in the United States. The Journal of School Nursing, 34(3), 232–244. 10.1177/1059840517752456 29343160

